# Current status and perspectives of the future of pancreatic surgery: Establishment of evidence by integration of “art” and “science”

**DOI:** 10.1002/ags3.12494

**Published:** 2021-08-05

**Authors:** Mee Joo Kang, Sun‐Whe Kim

**Affiliations:** ^1^ Department of Surgery Center for Liver and Pancreato‐Biliary Cancer National Cancer Center Goyang‐si Korea

**Keywords:** evidence‐based medicine, inventions, neoplasms, pancreas, pancreatectomy

## Abstract

Pancreatic cancer surgery continues to be associated with a high operative morbidity rate, poor long‐term survival outcomes, and various challenges in obtaining high‐level evidence. Not only is the early postoperative morbidity rate high, but also late morbidity involves lifelong nutritional support for long‐term survivors. Due to poor survival outcomes even after curative surgery, pancreatic surgeons have doubts about the role of surgery as the definitive treatment for pancreatic cancer. Additionally, conducting clinical trials to obtain high‐level evidence in the field of pancreatic surgery is difficult, and the results have only had a moderate impact on clinical practice due to skepticism regarding their quality. Therefore, quality evidence regarding the extent of resection, mode of approach to dissection, reconstruction methods for pancreatico‐enteric anastomosis, determination of resectability, timing of surgery, and the definition of the resection margin is lacking. However, numerous innovative pancreatic surgical procedures have been developed, which may aptly have been called “art” when they were first introduced, regardless of whether they subsequently were supported by scientific evidence. In this review, we provide recent examples of the integration of art and science in the field of pancreatic surgery, which illustrate how the creative ideas of pancreatic surgeons evolved into generally accepted clinical practice. Pancreatic surgeons should be considered “surgical artists,” “surgical scientists,” and “surgical practitioners.” We look forward to more “surgical artists” educating future “surgical artists and scientists” to create a richer “spirit of innovation,” leading to a more beautiful integration of art and science in the field of pancreatic surgery.

## Graphical abstract

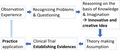

## 1 INTRODUCTION

**FIGURE 1:**
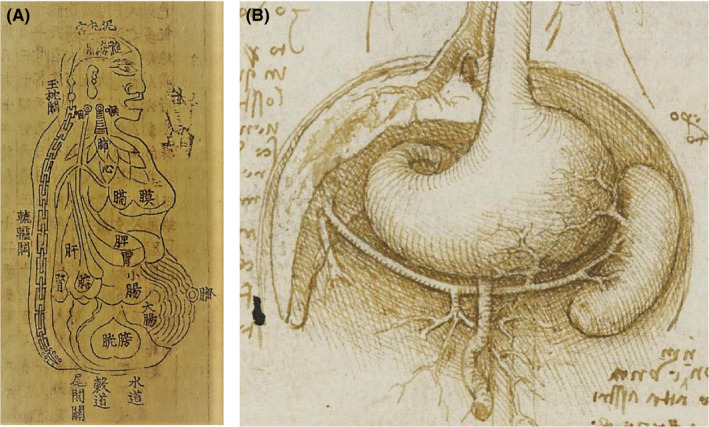
Understanding abdominal viscera as represented in classics from the Orient and the Occident. A. Drawing of the overall body, viscera, and bowel (Sinhyeongjangbudo) in Donguibogam, (Principles and Practice of Eastern Medicine; Memory of the World, UNESCO, Reproduced from Cultural Heritage Administration of the Republic of Korea according to Korea Open Government License, available from https://www.cha.go.kr/unescoGallery/selectUnescoGalleryView.do?id=189978). B. Abdominal anatomy according to Leonardo da Vinci (Reproduced from “Recto: The gastrointestinal tract and the bladder. Verso: The gastrointestinal tract, and the stomach, liver, and spleen c.1508” by Leonardo da Vinci with permission from the Royal Collection Trust, Royal Collection Trust/© Her Majesty Queen Elizabeth II 2021, available from https://www.rct.uk/collection/919031/recto‐the‐gastrointestinal‐tract‐and‐the‐bladder‐verso‐the‐gastrointestinal‐tract)

Compared to other malignancies of the gastrointestinal tract, pancreatic cancer surgery is unique in terms of its associated high operative morbidity rate, poor long‐term survival outcomes, and challenges in terms of obtaining high‐level evidence based on randomized clinical trials (RCTs). Until recently, the pancreas has been referred to as “no man's land,” which may explain why even the brightest thinkers in the East and West did not describe the pancreas in their anatomical diagrams (Figure 1).

After pancreatectomy, early morbidity is associated with pancreatic leakage, bleeding, delayed gastric emptying, and local sepsis, which often result from complex surgical procedures requiring multiple anastomoses.^1^, ^2^, ^3^ In addition, surgery‐related and systemic complications are more frequent due to the long operation time and, compared to other gastrointestinal malignancies, the general preoperative condition of patients with pancreatic cancer is worse. Late operative morbidity consists of exocrine and endocrine pancreatic insufficiency due to the loss of pancreatic parenchyma and marginal ulcers or the development of afferent loop syndrome attributable to gastrointestinal tract reconstruction.^4^ These late complications give rise to nutritional disorders demanding lifelong nutritional support in long‐term survivors.^5^ Therefore, the history of pancreatectomy procedures and treatments lead to the constant efforts currently made to reduce postoperative complications.

Moreover, survival outcomes for pancreatic cancer remain low and have not improved significantly over the past few decades.^6^ Only 20%‐30% of patients at the time of pancreatic cancer diagnosis are considered candidates for surgery, while 70%‐80% of patients eventually fail to receive curative treatment mainly due to systemic metastasis.^6^ Moreover, due to poor survival outcomes even after curative resection, pancreatic surgeons have doubts about the role of surgery as the definitive treatment for pancreatic cancer. For these reasons, pancreatic cancer is considered a systemic disease; however, surgeons should also focus on achieving safer and more complete local control of the tumor through surgery.

Nevertheless, there is a lack of high‐level evidence in the field of pancreatic surgery. Consequently, we do not yet have detailed guidelines for pancreatic surgical procedures, partly due to limitations in conducting RCTs, especially those focused on pancreatic cancer. Despite a noticeable increase in the quantity and quality of RCTs focused on pancreatic surgery, many are limited in their design and reporting, including selective reporting, limited assessment of long‐term effects, and the risk of small sample bias.^7^, ^8^ Specifically, given small differences in the expected effect of pancreatic surgery, the sample size can be “prohibitively large” while the number of pancreatic cancer patients is insufficient for case recruitment.^9^ Moreover, standardization of surgical techniques is difficult due to the complexity of the procedures.^10^ As a result of deep‐rooted skepticism, RCTs for pancreatic surgery have had only a moderate impact on daily clinical practice. For this reason, neither a synthesis of evidence nor a systematic review of filtered evidence is easily obtainable for pancreatic cancer surgery.^11^

## 2 CURRENT ISSUES IN PANCREATIC SURGERY

As previously mentioned, a number of issues regarding pancreatic surgical procedures, especially those for pancreatic cancer, still need to be addressed (Table 1). However, authoritative in‐depth guidelines, including those from the National Comprehensive Cancer Network (NCCN),^12^American Society of Clinical Oncology (ASCO),^13^ European Society for Medical Oncology (ESMO),^14^ Japan Pancreas Society (JPS),^15^ and the International Study Group for Pancreatic Surgery (ISGPS),^16^ have yet to be provided for pancreatic surgery. Each of the current issues in pancreatic surgery will be discussed below.

**TABLE 1:** Current issues in pancreatic surgery

**Extent of surgery**
Organ
Observation vs surgery
Excision vs pancreatectomy
Partial vs total pancreatectomy
Pancreaticoduodenectomy: standard Whipple vs pylorus preservation vs pylorus resection
Organ preservation (duodenum, spleen, or splenic vessels)
Lymph node dissection: standard vs extended
Nerve plexus dissection: preservation vs half‐circumferential removal vs 360° removal
Major vessels resection
Portal vein or superior mesenteric vein: technical amenability in relation to the first or second jejunal vein and duodenal inferior margin
Superior mesenteric artery: under trials in some centers
Common hepatic artery
Celiac artery: distal pancreatectomy with celiac artery resection (DPCAR)
Meso‐pancreas excision
Definition of the area
Level of dissection
**Mode of approach for dissection**
Various superior mesenteric artery first approach
Infra‐colic (mesenteric) vs supra‐colic
Right or left vs supra‐pancreatic
Radical ante‐grade modular pancreato‐splenectomy (RAMPS): anterior and posterior
No touch isolation technique
*En bloc* dissection
Minimally invasive surgery
**Reconstruction method (pancreatic anastomosis)**
Pancreaticojejunostomy vs pancreaticogastrostomy
Dunking/invaginating method vs duct‐to‐mucosa anastomosis
Internal vs external vs no stent
Many innovative modifications of pancreatic restoration
Usage and duration of surgical drain
**Transection and stump management**
Stapling vs hand sewing
With or without use of sealant, type of sealant
With or without use of somatostatin
**Determination of resectability**
Criteria
Various criteria: National Comprehensive Cancer Network (NCCN) criteria, etc.
International Association of Pancreatology (IAP) consensus criteria (+ biologic/conditional criteria)
Tools and their reliability: CT, MRI, PET, cytology, etc.
Determination after neoadjuvant treatment
Response evaluation by CT tumor marker, PET, etc.
**Timing of Surgery**
Upfront surgery, surgery after neoadjuvant therapy
Timing and indication of conversion surgery
**Resection margin and residual tumor**
Significance of multiple margins
Concept of residual tumor (tumor at the margin vs 1‐mm margin)

First, there is much debate regarding the appropriate extent of resection, which includes observation vs surgery, excision vs partial pancreatectomy, partial vs total pancreatectomy, the extent of gastric resection during pancreaticoduodenectomy ([PD]; standard Whipple operation, pylorus preservation, or pylorus resection), and organ preservation (duodenum, spleen, or splenic vessels; detailed in the “spleen‐preserving distal pancreatectomy” section), depending on the nature and extent of the disease.^17^, ^18^ Additionally, the extent of lymphadenectomy, resection of major vessels, and nerve plexus dissection can be controversial for several reasons; for example, whether to include the resection or dissection of remote site lymph nodes, the portomesenteric vein, and major arteries including the celiac axis (detailed in the “modified Appleby operation for advanced pancreatic body cancer” section), superior mesenteric artery (SMA), and hepatic arteries. Previous RCTs have confirmed that prophylactic hemi‐circumferential peri‐SMA nerve plexus dissection was not beneficial for survival gain.^19^, ^20^, ^21^, ^22^ However, even though R0 resection can be achieved by adjusting the dissection level, no consensus has been achieved on the systematic criteria for the extent of resection according to the extent of the patient's primary disease.^23^ Finally, although the definition remains controversial, the mesopancreas should also be considered when determining the extent of surgery (detailed in the “meso‐pancreas excision” section).^24^

Second, the various modes of approaching dissection require further discussion, which include different types of SMA (first) approaches mainly for pancreatic head cancer,^25^, ^26^, ^27^, ^28^, ^29^, ^30^, ^31^ radical ante‐grade modular pancreato‐splenectomy for pancreatic body and tail cancer,^32^, ^33^ the no‐touch isolation technique,^28^ *en bloc* dissection,^34^, ^35^ and minimally invasive surgery.^36^, ^37^, ^38^ Recently, the Miami International Evidence‐based Guidelines strongly recommended minimally invasive distal pancreatectomy for benign and low‐grade malignant tumors over open surgery; however, data on the advantages of minimally invasive PD over open surgery are insufficient. Minimally invasive pancreatectomy is recommended in high‐volume centers, and the requirement for a structured training program for minimally invasive pancreatectomy must also be emphasized.^38^

Third, a major factor contributing to morbidity after pancreatectomy is the leakage of pancreatic juice from the pancreatico‐enterostomy or pancreatic stump. Therefore, pancreatic surgeons are continuously searching for the best techniques for anastomosis and stump closure, which include various methods of reconstruction, the site and route of anastomosis, the use and type of stent or surgical drain, the use of surgical tissue adhesives, and other innovative techniques to restore pancreatico‐enteric continuity.^39^, ^40^

Fourth, the criteria and tools for determining resectability are constantly evolving. To date, several resectability criteria have been proposed by various institutions and academic societies. Since its first introduction in 2002, the NCCN resectability criteria have been the most widely adopted.^12^ Particularly, diagnostic criteria for borderline resectable pancreatic cancer have primarily evolved with a focus on technical resectability.^16^ However, the concept of this disease entity includes oncological curability supported by neoadjuvant treatment.^41^ Consequently, the criteria for determining tumor resectability are expanding beyond only anatomical or technical perspectives. Recently, the biological criteria proposed by the MD Anderson Cancer Center and the International Association of Pancreatology (IAP) have been validated.^42^, ^43^ Criteria for imaging and non‐anatomical criteria such as tumor markers, patient condition, and genetic profiling are also being developed.^44^ In particular, establishing criteria for biological markers and morphological and functional imaging after neoadjuvant therapy is challenging.^45^ Furthermore, the appropriate timing of surgery (upfront or after neoadjuvant therapy) and conversion surgery is fiercely debated.^46^

Lastly, the appropriate definition and assessment of the resection margin of the surgical specimen is controversial. PD specimens for pancreatic cancer have the most resection margins among the gastrointestinal malignancies, with seven margin evaluations recommended, including transection (pancreatic neck, proximal and distal gastrointestinal tract, bile duct, and vessel segment [if present]) and circumferential margins (portomesenteric groove, SMA margin [or retroperitoneal margin], posterior margin, and anterior surface).^12^ In addition, a consensus regarding the definition of “positive resection margin” has not been reached. In 1977, the American Joint Committee on Cancer defined a positive resection margin as the presence of tumor cells on the margin.^47^ However, the alternative definition presented in 2006 by the Royal College of Pathologists included the presence of tumor cells within 1 mm of the resection margin.^48^, ^49^ Since this new definition was introduced, the prognostic implications and real‐world practicality have been debated.^50^, ^51^

## 3 WHAT IS “ART,” “SCIENCE,” AND THEIR INTEGRATION?

Some of the abovementioned current issues have reached some level of consensus, while others are still under debate. As evidence accumulates over time, the level of consensus will likely increase in the future. However, by reflecting on past experiences, we can speculate on how this process will unfold in the future; the process required not only the mechanical accumulation of evidence, but also innovation and imagination that could be described as "art" beyond the boundaries of science.

**FIGURE 2:**
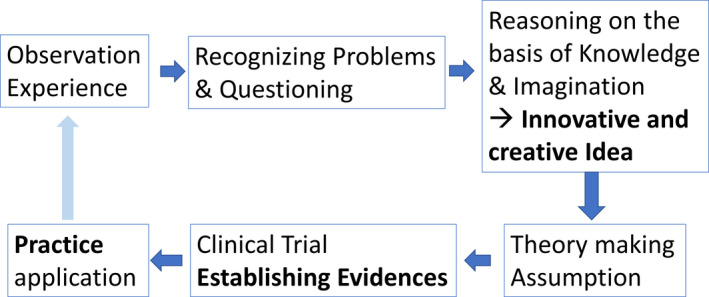
A sample diagram of the integration of art and science in the field of surgery

Based on these concepts, a sample diagram integrating “art” and “science” in the field of surgery is presented in Figure 2. Surgeons recognize problems and raise questions based on their observations and experiences in clinical practice. Surgeons’ reasoning based on their knowledge and imagination can lead to the development of innovative and creative ideas, which result in theories and hypotheses that enable surgeons to conduct RCTs to establish evidence for future clinical applications. This process exists as an iterative cycle because new practice cannot be perfect.

Most surgeons would claim they know what science is; however, what is art? The following are representative quotes about art that set it apart from science: “Art is not based on evidence, but on experience. Art does not confine to logic, it is an expression of feeling. Art does not work with hypothesis and does not need evidence and hence art is separate from science”^52^ and “art is the solution of a problem which cannot be expressed explicitly until it is solved. Art is the creative process and it goes through all fields.”^53^

There have been numerous creative and innovative surgical procedures in the history of pancreatic surgery that deserve to be called “art.” Most of the procedures belong to the realm of “art” when they are first introduced, regardless of whether they have been supported by scientific evidence or have become general practice. Likewise, there are many concepts and techniques applied to pancreatic surgery that are still considered “art.” Consequently, a few recent examples of “art, science, and their integration” are presented below. The first example demonstrates an experience in which an innovative technique, introduced by a need derived from clinical experience, continued to evolve through the integration of art and science. The second example illustrates how an innovative procedure adopted from other surgical disciplines overcame problems specific to pancreatic surgery. The third example shows the continuous integration of art and science regarding the meso‐pancreas, whose concept has not yet been fully established; the innovative concept, along with the development of a variety of surgical approaches in the area, is being evidenced through ongoing trials. The last example describes the integration of physiology and pancreatic surgery, which goes beyond the limits of the anatomical or technical perspective of surgery, often overlooked by surgeons.

### 3.1 Spleen‐preserving distal pancreatectomy

**FIGURE 3:**
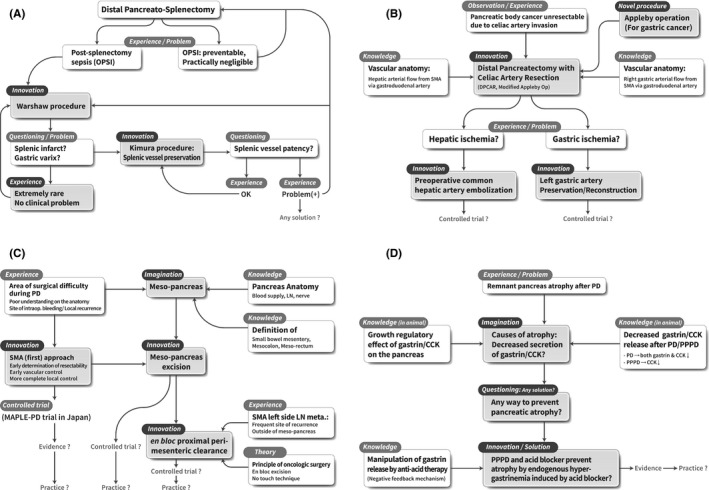
Cascade of knowledge, experience, questioning, hypothesis, and building evidence triggered by imagination and innovation. A. Spleen‐preserving distal pancreatectomy. B. Modified Appleby operation for advanced pancreatic body cancer. C. Meso‐pancreas excision. D. Preventing atrophy of the remnant pancreas after pancreatic head resection

Overwhelming post‐splenectomy infection (OPSI) is a rare but fatal complication after distal pancreatectomy with splenectomy (Figure 3a).^54^ The first solution introduced to manage this problem was the Warshaw procedure, in which the spleen is preserved (though not the splenic vessels) during distal pancreatectomy.^55^ However, many surgeons had concerns about splenic infarction and gastric varix, which can develop after splenic vessel resection.^55^, ^56^ Therefore, the Kimura procedure, which involves the preservation of both the spleen and splenic vessels, was proposed.^57^ Many surgeons have attempted both the Warshaw and Kimura procedures and have demonstrated their feasibility in clinical practice. Although initial reports appealed for improved technical reliability and safety compared to traditional distal pancreatectomy with splenectomy,^57^ a recent systematic review and meta‐analysis have revealed that the incidence of splenic infarction and secondary splenectomy associated with the Kimura procedure is significantly lower than that of the Warshaw procedure.^58^

However, OPSI were discovered to be more rare and preventable than expected, and splenic infarction and gastric varices were also rare, with little clinical significance.^59^, ^60^ Moreover, the benefits of splenic vessel preservation are not clear, since long‐term patency of the preserved vessels is uncertain.^61^ As a result, surgeons are currently performing all three procedures (conventional splenectomy and spleen preservation with or without splenic vessel preservation) based on clinical experience and value judgments regarding the risk of complications. As “practitioners,” surgeons can choose between these options based on their own experience, their feasibility, and on evidence provided by the “surgical scientists.”

### 3.2 Modified Appleby operation for advanced pancreatic body cancer

Pancreatic body cancers frequently infiltrate the celiac artery due to its proximity to the tumor. Originally designed for advanced gastric cancer, the Appleby operation includes a total gastrectomy and celiac artery resection (Figure 3‐b).^62^ The modified Appleby procedure, which involves preserving the right gastric artery and right gastroepiploic vessel to preserve the entire stomach, has been proposed and shown to be technically feasible as a treatment for pancreatic body cancer.^63^, ^64^ Additionally, hepatic and gastric blood flow is expected to be preserved by collateral blood flow from the SMA through the gastroduodenal and pancreaticoduodenal arteries after celiac and common hepatic artery resections. However, understandably, many pancreatic surgeons are concerned about hepatic and gastric ischemia, which have been reported but are extremely rare.^65^ Therefore, innovative procedures have been developed to address these issues, including preoperative common hepatic artery embolization to ensure hepatic blood flow^34^, ^66^ and the preservation or reconstruction of the left gastric artery to prevent gastric ischemia.^67^, ^68^ Since these innovative techniques have only been reported in a limited number of cases, RCTs are required to confirm their technical feasibility and safety.

### 3.3 Meso‐pancreas excision

Based on our understanding of pancreatic anatomy and the definition of the mesentery, meso‐colon, and meso‐rectum, the concept of the meso‐pancreas has emerged (Figure 3‐c).^69^ Although the anatomical concept of the meso‐pancreas is still controversial, some surgeons perform meso‐pancreas excision during PD for pancreatic head cancer to remove all soft tissue from this area since tumor involvement of retroperitoneal resection margin (R1 resection) and local recurrence is frequently reported after surgery.^70^, ^71^ In addition, innovative SMA‐first approaches that emphasize the principles of oncological surgery have been introduced to facilitate meso‐pancreas excision. One example is the MAPLE‐PD (mesenteric approach versus conventional approach for pancreatic cancer during Pancreaticoduodenectomy) trial currently in progress in Japan.^72^ Another example is the concept of “*en bloc* proximal peri‐mesenteric clearance,” which was proposed due to cancer recurrence at the SMA left‐side lymph node, located outside of the meso‐pancreas, and for which RCT‐based evidence is expected in the future.^35^ Through the above process, we can speculate how art and science are integrated to refine the establishment of innovative surgical procedures. All surgeons who have proposed this concept and attempted meso‐pancreas excision should be considered “surgical artists” as well as “surgical scientists.”

### 3.4 Preventing atrophy of the remnant pancreas after pancreatic head resection

Atrophy of the distal remnant pancreas is a frequently observed phenomenon after PD due to pancreaticojejunostomy stricture, postoperative radiation therapy, or the presence of an altered route for food passage, among others (Figure 3‐d).^73^ Additionally, the physiological change in gastrin/cholecystokinin (CKK) secretion has emerged as another potential cause of atrophy. This is based on the understanding that gastrin/CCK secretion is reduced after PD or pylorus‐preserving PD (PPPD) and through the growth‐stimulating effect that gastrin/CCK has on the pancreas that has clearly been identified in animal models.^74^ Therefore, it has been theorized that one of the causes of remnant pancreatic atrophy after PD/PPPD could be a decrease in gastrin/CCK secretion after PD and a decrease in CCK secretion after PPPD. Gastrin is secreted from the gastric antrum, and CCK is secreted from the duodenum; therefore, a consequence of both PD and PPPD is the removal of the source of these hormones.^75^

Therefore, we wondered if there was any way to prevent pancreatic atrophy. The extent of post‐PPPD atrophy was hypothesized to be less than that of post‐PD atrophy because PPPD preserves the gastrin secretion zone. In addition, a significant level of preserved gastrin/CCK response was observed in post‐PPPD patients but not in post‐PD patients.^76^, ^77^ Based on the knowledge that gastrin secretion can be manipulated by acid blockers through physiological negative feedback mechanisms,^78^ an innovative idea emerged; it was hypothesized that acid blockers may prevent or reduce the extent of atrophy of the remnant pancreas after PPPD by stimulating endogenous gastrin secretion. Subsequently, an RCT of patients after PPPD with induced hypergastrinemia showed a significant reduction in the extent of atrophy of the distal pancreas, along with an increased level of stool elastase.^73^ Although long‐term administration of acid blockers after PD has not become general practice due to potential side effects, it was an innovation that should be considered an “art,” since a solution was proposed from a new perspective based on the surgeon's observation and experience. Pancreatic exocrine insufficiency that occurs immediately after PD/PPPD in association with pancreatic tissue loss and gastrin/CCK regulation gradually returns to normal functional levels within 6 months.^4^, ^79^ Therefore, the current version of the ISGPS position paper recommends that pancreatic enzyme replacement therapy should be routinely initiated and continued for at least 6 months postoperatively in patients who undergo PD.^5^

## 4 CONCLUSION

This review illustrates how the creative ideas of pancreatic surgeons have evolved into generally accepted clinical practice. Surgeons should be considered “surgical artists,” “surgical scientists,” and “surgical practitioners” due to their capacity to combine art and science in clinical practice. We look forward to witnessing more “surgical artists” educating future “surgical artists and scientists” to continue the rich “spirit of innovation” in pancreatic surgery, which will lead to more innovative ideas and the development of more efficient methods of establishing high levels of evidence, and, thus, a more beautiful integration of art and science in the field of pancreatic surgery.

## DISCLOSURE

Funding: The authors received no specific funding for this study.

Conflict of interest: The authors have no conflicts of interest or financial ties to disclose.

Author Contribution: Mee Joo Kang: conceptualization (supporting), formal analysis (supporting), methodology (supporting), visualization (equal), writing – original draft preparation (equal), writing – review & editing (equal). Sun‐Whe Kim: conceptualization (lead), formal analysis (lead), methodology (lead), supervision (lead), visualization (equal), writing – original draft preparation (equal), writing – review & editing (equal).
